# A Compend of Obstetrics, Especially Adapted to the Use of Medical Students and Physicians

**Published:** 1899-06

**Authors:** 


A Compend of Obstetrics, Especially Adapted to the Use of Medical Students and Physicians By Henry G. Landis, A. M., M. D. Revised and edited by William H. Wells, M. D, Adjunct Professor of Obstetrics and the Diseases of Infancy in the Philadelphia Polyclinic, etc., etc. Sixth edition, illustrated. Quiz Compends No. 5. Philadelphia: P. Blakiston's Son & Co. 1898.

The editor has in this edition enlarged the chapters devoted to the diagnosis of the various positions and presentations by external methods, the mechanism of labor, the differential diagnosis between pregnancy and other forms of abdominal tumors, and obstetric operations. The subject of puerperal infection is also taught at greater length and in accordance with modern views. M. J. F.

